# Score interpretation thresholds for the European Organisation for Research and Treatment of Cancer thyroid cancer quality of life questionnaire (EORTC QLQ-THY34). An international prospective study

**DOI:** 10.1007/s12020-026-04835-w

**Published:** 2026-10-29

**Authors:** Susanne Singer, Gerasimos P. Sykiotis, Monica Pinto, Ioannis Iakovou, Laura Deborah Locati, Arild Andre Østhus, Eva Hammerlid, Rita Canotilho, Giuseppe Fanetti, Naomi Kiyota, Ricardo Ribeiro Gama, Matthias Büttner, Katherine J. Taylor, Juan Ignacio Arraras, Georgios Ioannidis, Orlando Guntinas-Lichius, Guy Andry, Olga Husson, Dagmar Führer, Marek Dedecjus, Silke Tribius, Akram Al-Ibraheem

**Affiliations:** 1 Comprehensive Cancer Center Mecklenburg-Vorpommern, University Medical Center Rostock, Rostock, Germany; 2 Service of Endocrinology, Diabetology and Metabolism, Lausanne University Hospital and University of Lausanne, Lausanne, Switzerland; 3 Rehabilitation Medicine Unit, Strategic Health Services Department, Istituto Nazionale Tumori – IRCCS – Fondazione G. Pascale, Naples, Italy; 4 Department of Nuclear Medicine, Aristotle University, Thessaloniki, Greece; 5 Department of Internal Medicine and Medical Therapeutics, Medical Oncology Unit, University of Pavia, Istituti Clinici Scientifici Maugeri IRCCS, Pavia, Italy; 6 ENT and Head and Neck Department, University Medical Centre Oslo, Oslo, Norway; 7 Institute of Clinical Sciences, Sahlgrenska Academy / Department of Otorhinolaryngology-Head and Neck Surgery, University of Gothenburg / Sahlgrenska University Hospital, Gothenburg, Sweden; 8 Head and Neck Surgery Department, Hospital das Clínicas, Faculdade de MedicinaInstituto Português de Oncologia do Porto Francisco Gentil, EPE, Porto, Portugal; 9 Division of Radiotherapy, Centro di Riferimento Oncologico di Aviano (CRO) IRCCS, (PN), Aviano, Italy; 10 Department of Medical Oncology and Hematology, Cancer Center, Kobe University Hospital, Kobe, Japan; 11 Barretos Cancer Hospital, Brazil; Head and Neck Surgery Department, Instituto do Câncer do Estado de São Paulo, Hospital das Clínicas, Faculdade de Medicina, Universidade de São Paulo (ICESP -HCFMUSP), Barretos, Brazil; 12 Department of Women’s and Children’s Health, Uppsala University, Uppsala, Sweden; 13 Medical Oncology Department, Hospital Universitario de Navarra, Pamplona, Spain; 14 Oncology Department, State Health Services Organization, Nicosia General Hospital, Nicosia, Cyprus; 15 Department of Otorhinolaryngology, Head and Neck Surgery, Jena University Hospital, Jena, Germany; 16 Surgery Department, Jules Bordet Institute, Brussels, Belgium; 17 Department of Medical Oncology, Netherlands Cancer Institute, Amsterdam, Netherlands, Departments of Public Health & Surgical Oncology, Erasmus University Medical Center Rotterdam, Netherlands, Amsterdam, Netherlands; 18 Department of Endocrinology, Diabetes and Metabolism, Centre for Endocrine Medicine (CEM), Member of ENDO-ERN and EURACAN, University Hospital Essen, University of Duisburg-Essen, Essen, Germany, Essen, Germany; 19 Department of Oncological Endocrinology and Nuclear Medicine, Maria Skłdowska-Curie Memorial National Research Institute of Oncology, Warsaw, Poland, Warsaw, Poland; 20 Asklepios Tumorzentrum Hamburg, Asklepios Klinik St. Georg, Hamburg, Germany, Hamburg, Germany; 21 Department of Nuclear Medicine, King Hussein Cancer Center, Amman, Jordan

**Keywords:** Minimal important change, Minimal important difference, Minimal detectable difference, Patient group-specific important differences, Clinical significance, Subjective significance, EORTC QLQ-C30, EORTC QLQ-THY34, Quality of life, Patient-reported outcomes

## Abstract

**Purpose:**

The European Organisation for Research and Treatment of Cancer Thyroid Cancer Quality of Life (QoL) Module (EORTC QLQ-THY34) is a valid tool to assess QoL in this patient population. The present study established estimates for score interpretation thresholds for this instrument when using it with thyroid cancer patients.

**Methods:**

Thyroid cancer patients under active treatment were contacted three times (t1: before treatment, t2: six weeks after t1, t3: six months after t2). They completed the EORTC QLQ-C30, the EORTC QLQ-THY34, and the Subjective Significance Questionnaire, indicating experienced change from the previous time point in five QoL domains. The Minimal Important Change (MIC) was established based on logistic regression models. Additionally, Minimal Detectable Change (MDC) was computed using 0.5 standard deviation and standard error of the measurement.

**Results:**

Altogether, 275 patients from 15 countries participated. MIC for deterioration and improvement were respectively 5 and 1 for *exhaustion*, 13 and 7 for *voice problems*, 11 and 2 for *tingling sensation*, 12 and 11 for *altered temperature tolerance*, and 2 and 2 for *overall QoL*. The MDC estimates for deterioration ranged from 7 (*physical functioning)* to 25 (*dyspnoea);* for improvement, it ranged from 7 (*physical functioning)* to 15, for *appetite loss*,* cramps*, and *altered temperature tolerance.*

**Conclusions:**

No single MIC or MDC can be applied to all EORTC QLQ-THY34 scales, and the MIC for improvement are generally smaller than those for deterioration. The estimates can be used in research and clinical practice to interpret QoL scores in thyroid cancer patients.

## Background

In clinical trials, patient-reported outcomes are used to estimate the treatment effects from the patients’ perspective, among them health-related quality of life (QoL). Usually, this is measured with standardised questionnaires [1] which can be generic (i.e., developed to cover QoL aspects that are relevant to many individuals) or specific (i.e., developed to measure QoL detriments that usually appear only after certain treatments or in certain diseases) or a combination of both. Most questionnaires contain several items that can be combined into scales, with the scores of these scales serving as trial endpoints.

However, what magnitude of score change is necessary to be considered clinically relevant? The answer to this important question is necessary for defining the sample size of a trial or to interpret the clinical benefit of an experimental treatment, but it is challenging to define [2]. Rules of thumb are frequently used, for example 10 points on a scale from 0 to 100 [3], but they have been found to be often insufficient: in different patient samples and for different scales, the Minimal Important Difference (MID) can be smaller or larger than 10 points, either due to clinical reasons or simply because the scoring only allows larger “jumps” [4]. Moreover, it has been shown that within individual scales thresholds for clinical importance often differ between improvement and deterioration [5–9]. This stems from patients accepting certain declines in their QoL before they consider them relevant, while even small improvements are appreciated as relevant [5]. Therefore, to obtain valid estimates of Minimal Important Change (MIC), it is necessary to empirically investigate the different scales of a given QoL instrument, and to do this separately for improvement and deterioration in each scale.

With this study, we aimed to determine such thresholds for clinical relevance for a newly developed instrument, the thyroid-cancer-specific module of the European Organisation for Research and Treatment of Cancer (EORTC), the EORTC QLQ-THY34 [10–13]. Based on suggestions from Terwee et al. [14], we use the following terminology:

- **Minimally Important Difference (MID)**: the smallest difference in QoL *between patient groups* that would lead to a change in treatment (including supportive care), based on an external criterion, an anchor.
- **Minimal Detectable Difference (MDD)**: the smallest difference in QoL *between patient groups* that can be statistically detected.
- **Minimally Important Change (MIC)**: refers to the smallest change in score that patients consider important.
- **Minimal Detectable Change (MDC)**: the smallest difference in QoL *within patients over time* that can be statistically detected.

There is ongoing debate about the equivalence of these concepts, with some authors naming all of them “minimal important difference” [9, 15, 16] whereas others argue that the minimal detectable difference and change are relevant thresholds but they should not be called “important” [14]. There are also suggestions to combine and integrate these approaches [16, 17]. In the present study, to ensure consistency with established terminology in the literature, we assess all of them (MIC, MDC, MID and MDD). According to current guidance [18], we consider anchor-based estimates (MIC/MID) to be more relevant clinically. As an umbrella term, we use “score interpretation thresholds” (SIT) [19, 20] as the concept encompassing the MID, MDD, MIC and MDC.

One of the most widely used questionnaires to assess QoL in oncology is the EORTC Core Questionnaire, the EORTC QLQ-C30 [1, 21] for which estimates for the MID [22–29] and MIC [6–9, 30] have been published. However, for its various disease- and condition-specific modules, only a few papers have investigated these thresholds. SIT are available for the pancreatic cancer [31], the myeloma [32], the brain cancer [33], and for the head and neck cancer module [7, 8].

In this paper, we present SIT estimates for the EORTC QLQ-THY34 as well as the EORTC QLQ-C30 specifically for patients with thyroid cancer.

## Methods

### Study design and data collection

This was a multi-national prospective cohort study with consecutive accrual. Its aim was to determine key psychometric properties of the EORTC QLQ-THY34 [12]. Patients included were 16 years or older, able to complete a questionnaire in the local language and had a cytologically or histologically verified diagnosis of thyroid cancer (papillary, follicular, oncocytic, poorly differentiated, medullary, anaplastic, and mixed). Exclusion criteria were concurrent malignancies (i.e., any ICD-10 C diagnosis except non-melanoma skin cancer) and participation in previous phases of the instrument’s development. For the current analyses, we used data from patients who were about to start treatment or best supportive care (Group 1), because this group was expected to experience changes in their QoL. Group 2 included survivors ≥ 2 years after diagnosis without structural evidence of disease (based on imaging) and without anti-neoplastic treatment within the last 12 months for whom QoL was expected to be relatively stable. Their data were used to define test-retest reliability.

Patients completed the questionnaires before treatment or best supportive care started (baseline - t1), 6 weeks after the first day of treatment (t2), and six months after the second time-point (t3). We assumed that QoL would deteriorate in most patients after t1 and improve again until t3, based on the literature [34–37] and the investigators’ clinical experience.

### Instruments

We estimated the SIT for the EORTC QLQ-C30 [21] and its thyroid cancer module, the EORTC QLQ-THY34 [12]. The EORTC QLQ-C30 consists of 30 items, combined into five functional and nine symptom scales. The EORTC QLQ-THY34 has 17 scales; these are: *exhaustion (EX)*,* discomfort in the head and neck (DI)*,* voice (VO)*,* hair problems (HA)*,* swallowing (SW)*,* treatment- and disease-related worry (TD)*,* tingling or numbness (TI)*,* worry about important others (WO)*,* lacking social support (SO)*,* dry mouth (DM)*,* altered temperature tolerance (TO)*,* body image (BI)*,* shoulder functioning (SH)*,* joint pain (JP)*,* cramps (CR)*,* impact on job or education (JE)*, and *rapid heartbeat (RH).*

A custom Subjective Significance Questionnaire (SSQ) [3], completed at t2 and t3, served as anchor for important change. To keep the questionnaire from being lengthy, we did not ask participants to assess changes in their QoL in all domains but only those domains which had emerged as the most important in our phase I study [10]. In addition, overall quality of life was included due to its general applicability. Of note, the respective scale of the EORTC QLQ-C30 is named “global health status/quality of life” but for the SSQ, we used the original wording developed by Osoba et al. [33] which is “overall quality of life”. With the SSQ, the patients rated the extent to which their QoL had worsened or improved in the following domains: *fatigue*,* voice*,* tingling sensations*,* temperature tolerance* and *overall quality of life* compared to the previous time point (e.g., “Since the last time I filled out the questionnaire, my fatigue is .”). The response options ranged from very much worse to very much better on a 7-point Likert scale, following the response format used by Osoba et al. [3].

### Analysis

The statistical analysis plan was developed based on our experience with calculating SIT for the EORTC head and neck module [7, 8] as well as on guidance from Dutch [14, 38–40], Canadian [41], and US American [16] colleagues.

#### Descriptive analyses

We calculated the mean change (delta) in EORTC QLQ-THY34 and EORTC QLQ-C30 scale scores between t1 and t2 as well as between t2 and t3, together with respective 95% confidence intervals. These are henceforth called the “EORTC change scores”. We then computed the percentage of (subjectively) deteriorated and improved patients based on the SSQ and plotted the distribution of EORTC change scores in the deteriorated and improved group.

#### Methods for estimating the minimal important difference (MID)

To determine valid anchors, our approach was as follows: (1) for each scale of the EORTC QLQ-THY34, an external criterion was suggested as a potential anchor by the first author, (2) four clinicians independently commented on its usefulness and practicability and suggested potential thresholds, (3) the criteria were then discussed among the investigators at an EORTC Quality of Life Group meeting and it was decided that all of the suggested criteria should be explored, (4) the correlation coefficients of the criteria with the scale scores at t2 and t3 were computed. We employed point biserial correlation for dichotomous variables and Spearman correlation for continuous variables. We predefined a correlation threshold of ≥ 0.70 to identify strong anchors, reflecting a conservative approach to strengthen validity and minimise the risk of spurious associations [41].

#### Methods for estimating the minimal detectable difference (MDD)

For all scales, we calculated (1) the standard error of measurement (SEM) of the respective scale score at t1, t2 and t3 using the formula SEM = SD * square root of (1-reliability) and the intra-class correlation coefficient between t1 and t2 as the reliability measure [40]; and (2) the 0.5 standard deviation of the respective scale score at t1, t2 and t3. We then computed the average of all 6 values per scale, which was considered to be the MDD estimate.

#### Methods for estimating the minimal important change (MIC)

The SSQ was used as an anchor for the determination of the MIC. The analyses differed by scale, because we had asked for specific changes in only five domains (*fatigue*,* voice*,* tingling sensations*,* temperature tolerance*,* overall QoL*). These are called *specific anchors* in the following. For all other scales, the global anchor* change in overall QoL* was used. The procedure was as follows:

##### MIC for deterioration

The responses per SSQ item measured at t2 were grouped into *deteriorated* (including the responses “very much worse”, “moderately worse”, “a little worse”) versus *not deteriorated* (including “about the same”, “a little better”, “moderately better”, “very much better”). Based on this, we calculated a logistic regression on deterioration between t1 and t2, with the EORTC change score between t1 and t2 as independent variable, and obtained the MIC based on predictive modelling (the MIC_predict_) by calculating (ln(odds-pre)-intercept)/regression coefficient [38].

##### MIC for improvement

SSQ responses from t3 were grouped into *improved* (“very much improved” to “a little improved”) versus *not improved* (“about the same” and all indications of worsening). Predictive modelling was performed analogous to the procedure for deterioration, but this time with improvement at t3 as outcome variable and the changes between t2 and t3 taken as covariate.

Since standard predictive modelling may be biased by the proportion of improved patients in a sample, if the percentage of changed patients was < 40%, the MIC_predict_ was corrected with the formula MIC_adjusted_ = MIC_predict_ - (0.09 + 0.103 x point biserial correlation coefficient of change score with anchor) × SD of change score x log-odds(imp) [14, 39]. To quantify the uncertainty of the MIC_predict_ estimates, we derived 95% confidence intervals using case-resampling bootstrap (2,000 replications, percentile method), whereby the full estimation procedure, including the correction applied when the proportion of changed patients was below 40%, was repeated within each bootstrap sample.

For scales without a specific anchor, we used the SSQ item *change in overall QoL* as the anchor. We did this for all scales (including the ones where a specific anchor was available, for comparison) but only as an ancillary analysis. We expected it to be a poor anchor because it is not specific.

Finally, we calculated point biserial correlations of EORTC change scores with the anchors. If the correlation coefficient was lower than 0.15, it was considered to be poor. We used different correlation thresholds for MID (≥ 0.70) and MIC (> 0.15) because the two constructs address different questions. The MID concerns a between-group difference that should be considered universally important; a weak anchor here risks conflating measurement noise with a genuine group difference, which justifies a stringent correlation requirement. The MIC, by contrast, concerns a within-patient change that patients themselves already reported as important; here the anchor’s role is descriptive rather than confirmatory, characterising the distribution of change scores among patients who reported an important change, rather than establishing that the change itself is genuine. A weaker anchor-scale correlation is therefore acceptable for MIC, though it does mean that MIC estimates derived from very weak anchors should be interpreted with more caution (as flagged in Tables 4 and 6).

#### Methods for estimating the Minimal Detectable Change (MDC)

For all scales, we determined distribution-based values for MDC and computed:

1. the SEM and the 0.5 SD of the delta between t1 and t2 to determine MDC for *deterioration*.
2. the SEM and the 0.5 SD of the delta between t2 and t3 to determine MDC for *improvement*.

The 0.5 SD was chosen to enable comparison with the literature [17].

## Results

### Sample

Patients were enrolled across 19 centres in the following 15 countries: Austria, Belgium, Brazil, Cyprus, Germany, Greece, Italy, Japan, Jordan, Netherlands, Norway, Portugal, Spain, Sweden, and Switzerland.

**Fig. 1:**
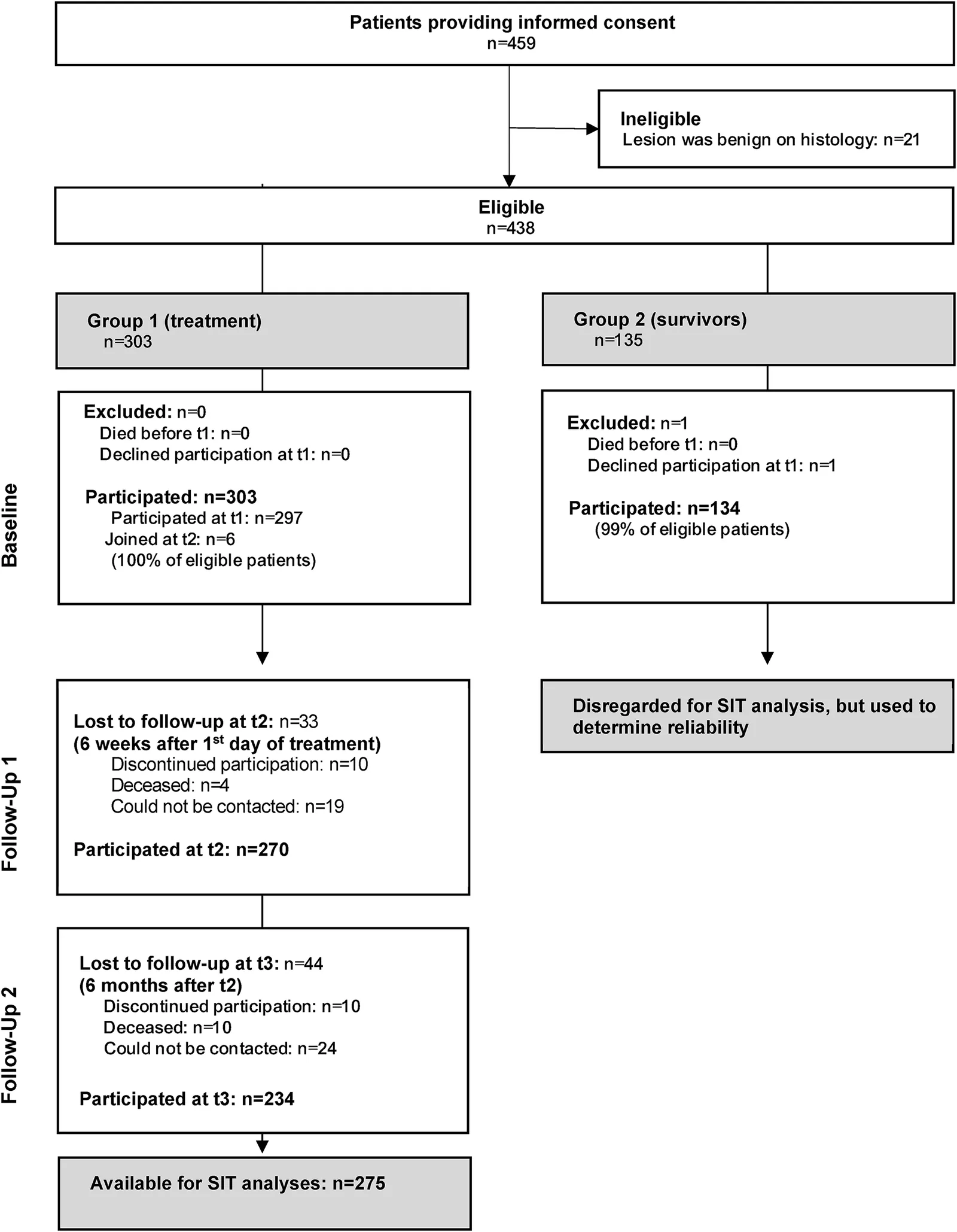
Patient flow through the study

A total of 459 patients were screened, of whom *n* = 438 were eligible and *n* = 437 participated. Of those, *n* = 134 (Group 2) were used to determine test-retest reliability, already published [12]. They were not suitable for estimating MIC and MDC because no clinically relevant change in QoL was expected and were thus disregarded for the current analysis. Of the patients in Group 1, about to undergo treatment or best supportive care, *n* = 275 participated at least twice, making them suitable for the SIT analyses (see Flowchart in Fig. 1). The majority (71%) of these 275 participants were female and had papillary thyroid cancer (Table 1 for more details). Most of the patients (69%) received multimodal treatment, 30% received monotherapy, and four patients (1%) received none of surgery, RAI, radiotherapy, TKI, or chemotherapy (for more details on the distribution of treatment combinations see eTable 1). One patient was younger than 18 years.

**Table 1:** Demographic and clinical characteristics of the participants (*n* = 275)

Variable	Category	*N* (%)
**Gender**	Male	80 (29.1%)
	Female	195 (70.9%)
**Age**	< 40 years	94 (34.2%)
	40–49 years	48 (17.5%)
	50–59 years	48 (17.5%)
	60–69 years	47 (17.1%)
	70–79 years	33 (12.0%)
	80 + years	5 (1.8%)
**Education**	< 10 years	52 (18.9%)
	10 years	33 (12.0%)
	> 10 years	187 (68.0%)
	Missing information	3 (1.1%)
**Country**	Italy	65 (23.6%)
	Jordan	41 (14.9%)
	Switzerland	36 (13.1%)
	Greece	25 (9.1%)
	Norway	20 (7.3%)
	Sweden	17 (6.2%)
	Austria	15 (5.5%)
	Germany	13 (4.7%)
	Portugal	10 (3.6%)
	Japan	9 (3.3%)
	Brazil	9 (3.3%)
	Spain	7 (2.5%)
	Cyprus	5 (1.8%)
	Belgium	2 (0.7%)
	The Netherlands	1 (0.4%)
**Histology**	Papillary	194 (70.5%)
	Follicular	22 (8.0%)
	Hürthle-cell	10 (3.6%)
	Poorly differentiated	6 (2.2%)
	Medullary	24 (8.7%)
	Anaplastic	15 (5.5%)
	Other	4 (1.5%)
**UICC stage**	I	151 (54.9%)
	II	40 (14.5%)
	III	19 (6.9%)
	IV	52 (18.9%)
	Missing information	13 (4.7%)
**ATA response to therapy** **(at t2, only for patients with DTC)**	No evidence of disease	99 (36.0%)
	Indeterminate	15 (5.5%)
	Biochemically incomplete	5 (1.8%)
	Structural disease	32 (11.6%)
	Unknown	80 (29.1%)
	Missing information	1 (0.4%)
**Treatment between enrolment and first follow-up** ***(several options possible)***	Partial thyroidectomy	19 (6.9%)
	Total thyroidectomy	81 (29.5%)
	Neck dissection central	54 (19.6%)
	Neck dissection lateral	18 (6.5%)
	Neck dissection central & lateral	63 (22.9%)
	Radioiodine for ablation	101 (36.7%)
	Radioiodine for therapy	47 (17.1%)
	Radiotherapy for local control	10 (3.6%)
	Radiotherapy for distant metastases	8 (2.9%)
	Tyrosine kinase inhibitors	30 (10.9%)
	Chemotherapy	7 (2.5%)
**Detection of cancer**	Because of symptoms	66 (24.0%)
	Because of neck lump	104 (37.8%)
	Incidental within surgery for goitre	10 (3.6%)
	Incidental on imaging	65 (23.6%)
	Other	21 (7.6%)
	Not yet determined at baseline	9 (3.3%)

*Note: UICC = Union Internationale Contre le Cancer; ATA = American Thyroid Association (the “unknown” category was used for situations in which the patient’s disease and treatment course did not yet permit classification); DTC = Differentiated Thyroid Carcinoma*

A comparison of patients by frequency of participation (eTable 2, eTable 3) showed that sex, UICC stage, ATA response to therapy at baseline, and planned treatment modality differed between those who completed one, two, or three assessments; however, these differences did not follow a simple gradient of disease severity, and were partly driven by categories reflecting missing or unclear clinical documentation rather than disease status itself. Age and histology did not differ, and, importantly, baseline QoL was similar across participation groups, suggesting that attrition was not associated with differences in QoL at study entry.

### Changes in quality of life and proportion of patients with improvement / deterioration

The mean changes in QoL in the various domains over time were generally modest and not always in the hypothesised direction (eTable 4 and eTable 5). Notably, the expected pattern of initial deterioration followed by recovery was not consistently observed across all domains, indicating heterogeneous patient experiences during treatment.

The proportion of patients who reported feeling a deterioration between t1 and t2 ranged from 18% (*tingling sensations*) to 29% (*fatigue*); the proportion who experienced improvement between t2 and t3 ranged from 29% (*temperature tolerance*) to 50% (*overall quality of life*) (Table 2).

**Table 2:** Proportion of patients who reported change in their quality of life

	Expected deterioration (t2 vs. t1) *n* = 270		Expected improvement (t3 vs. t2) *n* = 234	
**Specific anchor**	**Reported deterioration**	**Reported improvement**	**Reported deterioration**	**Reported improvement**
Fatigue	29%	38%	24%	49%
Voice	25%	27%	12%	39%
Tingling sensations	18%	32%	16%	38%
Temperature tolerance	19%	24%	17%	29%
Overall Quality of Life	26%	39%	24%	50%

*Note: Proportions of change were ascertained using the Subjective Significance Questionnaire (SSQ). For each domain*,* reported deterioration includes all patients who said their quality of life in the respective domain was very much worse*,* moderately worse*,* or a little worse compared to the previous time-point*,* and reported improvement includes all patients who said their quality of life was very much better*,* moderately better*,* or a little better. t1: before treatment; t2: six weeks after starting treatment; t3: six months after t2.*

### Minimal important difference (MID)

The correlation coefficients of the EORTC scales with the predefined external criteria turned out to be all far below the predefined threshold of 0.70 (eTable 6) which is why no MID could be calculated.

### Minimal detectable difference (MDD)

The MDD values, taken as the mean of SEM and SD-based estimates of all three time points combined, ranged from 7, for *nausea and vomiting*, to 17, for *dyspnoea* (Table 3).

**Table 3:** Minimal detectable difference (MDD) estimates

	t1		t2		t3		MDD
EORTC QLQ-THY34	SEM	0.5 SD	SEM	0.5 SD	SEM	**0.5 SD**	**Av**
Exhaustion	12	14	11	13	12	14	**13**
Discomfort in the Head and Neck	11	12	10	11	10	11	**11**
Voice	10	14	10	14	8	12	**12**
Hair Problems	6	13	7	15	7	15	**10**
Swallowing	7	10	7	10	7	10	**9**
Treatment- and Disease-related Worry	7	12	7	12	7	13	**9**
Tingling or Numbness	7	10	7	10	6	10	**8**
Worry about Important Others	8	15	7	14	8	15	**11**
Lacking Social Support	12	12	11	12	12	12	**12**
Dry Mouth	9	15	9	15	9	15	**12**
Altered Temperature Tolerance	13	16	13	15	13	16	**14**
Body Image	13	16	12	16	12	16	**14**
Shoulder Functioning	9	14	10	14	9	14	**12**
Joint Pain	12	16	12	16	12	15	**14**
Cramps	14	14	13	13	15	14	**14**
Impact on Job or Education	15	18	13	16	14	17	**15**
Rapid Heartbeat	11	11	11	11	12	12	**11**
EORTC QLQ-C30							
Physical Functioning	6	12	6	11	5	10	**8**
Role Functioning	12	17	11	15	10	14	**13**
Emotional Functioning	9	13	9	13	9	13	**11**
Cognitive Functioning	5	13	5	12	6	14	**9**
Social Functioning	9	14	9	13	9	13	**11**
Global Health Status/QoL	8	12	7	10	8	11	**9**
Fatigue	10	15	9	13	9	14	**12**
Nausea and Vomiting	8	8	8	8	7	7	**7**
Pain	12	15	11	14	11	14	**13**
Dyspnoea	23	25	13	14	13	14	**17**
Insomnia	13	18	12	16	12	16	**14**
Appetite Loss	15	13	15	12	15	13	**14**
Constipation	11	16	10	15	10	15	**13**
Diarrhoea	8	8	10	10	9	9	**9**
Financial Difficulties	7	14	7	14	7	14	**11**

*Note: SEM = Standard error of the measurement*,* SD = Standard deviation*,* Av = Average (mean) of both SEM- and SD-based MDD estimates of all three time-points; t1 = before treatment*,* t2 = six weeks after t1*,* t3 = six months after t2.*

### Minimal important change (MIC)

The correlation coefficients between the five *specific SSQ anchors* (i.e., *fatigue*,* voice*,* tingling sensations*,* temperature tolerance*,* overall quality of life*) and the respective EORTC change scores ranged from 0.35 to 0.48 regarding deterioration, and from 0.03 to 0.32 regarding improvement (Tables 4 and 6). The strongest correlation in both cases were between the *voice* anchor and the *voice* scale. The correlation with the *global SSQ anchoroverall quality of life* was ≥ 0.15 for 14 of the EORTC change scores for “deterioration” at t2 and for 12 EORTC change scores for “improvement” at t3; it was below this threshold in the other scales, suggesting that *overall quality of life* may not be a suitable anchor for many of the EORTC scales.

**Table 4:** Minimal important change (MIC) estimates

		Deterioration			Improvement		
	EORTC scale	Corr	MIC	95% CI	Corr	MIC	95% CI
**Specific Anchor**	Exhaustion	0.39	**5**	(-3; 13)	0.21	1	(-21; 21)
	Voice	0.48	**13**	(5; 22)	0.32	**7**	(-17; 3)
	Tingling or Numbness	0.39	**11**	(4; 19)	0.17	**2**	(-119; 114)
	Temperature Tolerance	0.28	**12**	(-4; 28)	0.03	**11**	(-463; 342)
	Global Quality of Life	0.35	**2**	(-8; 7)	0.27	2	(-7; 13)
**Global Anchor**	**EORTC QLQ-THY34**						
	Exhaustion	0.28	**3**	(-8; 16)	0.23	1	(-19; 16)
	Discomfort in the Head and Neck	0.30	**5**	(-6; 15)	0.11	6	(-62; 41)
	Voice	0.21	**8**	(-15; 31)	0.18	6	(-37; 17)
	Hair Problems	0.09	**5**	(-158; 117)	0.10	1	(-106; 106)
	Swallowing	0.25	**4**	(-8; 16)	0.04	4	(-196; 225)
	Treatment- and Disease-related Worry	0.10	**1**	(-79; 92)	0.23	0	(-12; 12)
	Tingling or Numbness	0.12	**5**	(-58; 57)	0.02	6	(-315; 219)
	Worry about Important Others	0.03	**6**	(-249; 247)	0.17	2	(-27; 21)
	Lacking Social Support	0.03	**5**	(-264; 320)	0.02	1	(-265; 330)
	Dry Mouth	0.11	**1**	(-119; 84)	0.02	7	(-382; 368)
	Altered Temperature Tolerance	0.18	**6**	(-21; 40)	0.02	1	(-431; 495)
	Body Image	0.15	**7**	(-30; 46)	0.23	2	(-26; 17)
	Shoulder Functioning	0.14	**4**	(-32; 53)	0.12	0	(-66; 49)
	Joint Pain	0.11	**5**	(-72; 90)	0.10	3	(-152; 97)
	Cramps	0.14	**2**	(-32; 48)	0.14	1	(-65; 40)
	Impact on Job or Education	0.01	**33**	(-348; 300)	0.21	2	(-22; 22)
	Rapid Heartbeat	0.04	**1**	(-239; 226)	0.12	0	(-74; 61)
	**EORTC QLQ-C30**						
	Physical Functioning	0.29	**2**	(-10; 6)	0.17	2	(-16; 25)
	Role Functioning	0.31	**3**	(-15; 11)	0.08	6	(-216; 220)
	Emotional Functioning	0.29	**2**	(-9; 12)	0.19	0	(-18; 24)
	Cognitive Functioning	0.23	**1**	(-14; 11)	0.10	2	(-86; 130)
	Social Functioning	0.33	**5**	(-14; 4)	0.16	3	(-27; 43)
	Global Health Status/QoL	0.35	**2**	(-8; 7)	0.27	2	(-7; 14)
	Fatigue	0.37	**8**	(-1; 18)	0.17	2	(-33; 23)
	Nausea and Vomiting	0.15	**1**	(-31; 32)	0.05	1	(-199; 155)
	Pain	0.21	**4**	(-25; 29)	0.19	1	(-30; 21)
	Dyspnoea	0.02	**27**	(-314; 310)	0.14	3	(-65; 33)
	Insomnia	0.12	**2**	(-77; 106)	0.13	3	(-105; 51)
	Appetite Loss	0.28	**3**	(-11; 16)	0.11	1	(-117; 114)
	Constipation	0.02	**17**	(-359; 380)	0.04	5	(-319; 252)
	Diarrhoea	0.11	**4**	(-65; 77)	0.06	1	(-249; 184)
	Financial Difficulties	0.06	**6**	(-148; 162)	0.08	2	(-160; 148)

Note: Displayed are the absolute values of the minimal important change scores based on predictive modelling (MIC_predict_) and the absolute values of the point biserial correlation between the EORTC change score and the anchor (Corr). Orange shading marks insufficiently strong association (threshold r ≤ 0.15). In these cases, the MIC should be used with caution. The MIC_predict_ values that were adjusted because the prevalence of change was below 40% are printed in green. 95% confidence intervals (CI) for MIC_predict_ were derived via bootstrap (2,000 replications, percentile method). Wide confidence intervals reflect the weak anchor-scale correlation for these domains and resulting instability of the MIC_predict_ estimate in individual bootstrap samples. Note that the MIC_predict_ is displayed as an absolute value because the direction of a “positive” change is not consistent across scales (e.g., a higher score indicates worsening on symptom scales such as Fatigue, but improvement on functioning scales such as Voice); absolute values therefore allow direct comparison of MIC magnitude across scales. The corresponding 95% CIs are reported on the original (signed) scale, i.e., prior to taking the absolute value of the point estimate, which is why the CI does not always numerically contain the (unsigned) point estimate shown

**Fig. 2:**
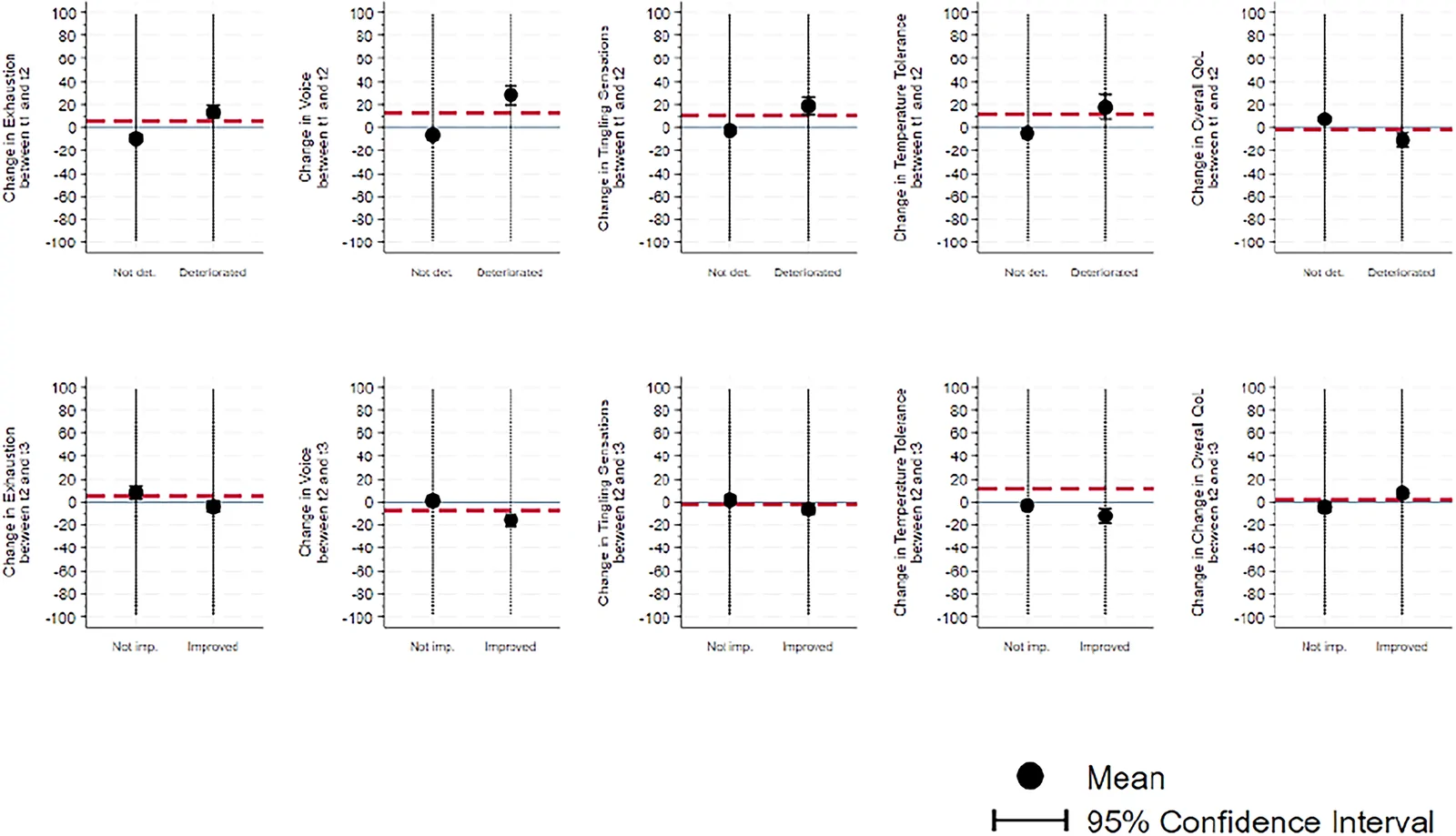
Distribution of EORTC change scores per Subjective Significance Questionnaire group (deteriorated vs. not deteriorated and improved vs. not improved). Notes: The EORTC change scores represent the difference in the score for the particular EORTC QLQ scale from the current time point compared to the previous one. Change scores >0 in the Exhaustion, Voice, Tingling Sensations, and Temperature Tolerance scales and <0 in Global Quality of Life represent a deterioration of quality of life, and vice versa. The dashed lines represent the minimal important change score based on predictive modelling (MICpredict). t1 = before treatment, t2 = six weeks after t1, t3 = six months after t2.

The MIC for deterioration and improvement were respectively 5 and 1 for *exhaustion*, 13 and 7 for *voice*, 11 and 2 for *tingling or numbness*, 12 and 11 for *temperature tolerance* (noting the low correlation between EORTC change score and anchor in this scale), and 2 and 2 for *global quality of life*. Overall, the MIC estimates based on specific anchors differentiated well between the patient groups (Fig. 2).

Ancillary analyses used the *overall quality of life* SSQ item as a global anchor to explore the MIC for all other EORTC scales. Here, the MIC for *deterioration* ranged from 1, for *nausea and vomiting*,* rapid heartbeat*, and *treatment- and disease-related worry*, to 33, for *impact on job or education*. The MIC for *improvement* ranged from 0, for *emotional functioning*, *shoulder functioning*, and *rapid heartbeat* to 7, for *dry mouth*. As noted, many of the correlations of the EORTC change scale with the global anchor were poor, and this approach was considered suboptimal.

### Minimal detectable change (MDC)

The minimal detectable EORTC change scores, taken as the average of SEM and SD-based estimates, for *deterioration* ranged from 7, for *physical functioning*, to 25, for *dyspnoea*. For *improvement*, it ranged from 7, for *physical functioning*, to 15, for *appetite loss*,* cramps*, and *altered temperature tolerance* (Table 5).

**Table 5:** Minimal detectable change (MDC) estimates

		Deterioration			Improvement		
**Scale**	**ICC**	**SEM**	**0.5 SD**	**Av**	**SEM**	**0.5 SD**	**Av**
**EORTC QLQ-THY34**							
Exhaustion	0.83	11	13	**12**	11	14	**13**
Discomfort in the Head and Neck	0.80	10	11	**11**	8	9	**9**
Voice	0.88	11	15	**13**	9	13	**11**
Hair Problems	0.95	6	13	**10**	6	14	**10**
Swallowing	0.87	8	11	**9**	7	9	**8**
Treatment- and Disease-related Worry	0.92	6	10	**8**	6	10	**8**
Tingling or Numbness	0.90	7	11	**9**	7	11	**9**
Worry about Important Others	0.93	7	13	**10**	6	12	**9**
Lacking Social Support	0.76	12	12	**12**	13	13	**13**
Dry Mouth	0.91	9	15	**12**	8	14	**11**
Altered Temperature Tolerance	0.83	13	16	**15**	14	17	**15**
Body Image	0.85	12	15	**13**	13	16	**14**
Shoulder Functioning	0.88	10	15	**12**	9	12	**10**
Joint Pain	0.85	12	15	**13**	12	16	**14**
Cramps	0.72	15	14	**15**	16	15	**15**
Impact on Job or Education	0.83	12	15	**14**	13	16	**14**
Rapid Heartbeat	0.76	11	11	**11**	12	12	**12**
**EORTC QLQ-C30**							
Physical Functioning	0.93	5	10	**7**	5	10	**7**
Role Functioning	0.87	12	16	**14**	11	15	**13**
Emotional Functioning	0.89	9	13	**11**	9	13	**11**
Cognitive Functioning	0.96	5	11	**8**	5	12	**8**
Social Functioning	0.88	8	12	**10**	9	14	**11**
Global Health Status/QoL	0.87	8	11	**10**	8	11	**9**
Fatigue	0.89	9	14	**12**	8	13	**11**
Nausea and Vomiting	0.77	10	10	**10**	8	9	**9**
Pain	0.85	12	16	**14**	11	14	**12**
Dyspnoea	0.79	23	26	**25**	12	14	**13**
Insomnia	0.86	13	17	**15**	12	17	**14**
Appetite Loss	0.66	17	14	**16**	16	14	**15**
Constipation	0.89	10	15	**12**	10	15	**12**
Diarrhoea	0.74	11	11	**11**	13	12	**12**
Financial Difficulties	0.94	5	11	**8**	6	13	**9**

*Note: ICC = Intra-class correlation coefficient*,* SEM = Standard error of measurement*,* SD = Standard deviation. Av = Average (mean) of both SEM- and SD-based values*,* constituting the MDC estimate*

## Discussion

The aim of this study was to establish thresholds for interpreting score differences of the EORTC QLQ-C30 and EORTC QLQ-THY34 questionnaires, which are frequently used worldwide for measuring health-related QoL in patients with thyroid cancer [37, 42–54]. There is considerable interest among clinicians and researchers in the development of such thresholds because they can aid in interpreting differences between patient groups and changes over time.

However, although the purpose of SIT is clear and its application straightforward, defining these estimates can be methodologically challenging unless only distribution-based approaches are used, which is considered to be suboptimal [18, 40]. To enhance the validity of our study, we relied on a range of methodological approaches, which we have tested and successfully applied before with the EORTC QLQ-HN43 [7, 8] as well as on data from a large cohort of thyroid cancer patients from 15 different countries who were prospectively surveyed.

One main finding is that specific anchors are better suited for determining MIC as they are more closely associated with the change in QoL in the specific domain. An important consideration is the strength of the association between anchors and change scores. While specific anchors performed better than the global anchor, several correlations were modest, particularly for improvement. Therefore, MIC estimates based on weaker anchors should be interpreted with caution. This is reflected in the width of the corresponding confidence intervals: while the MIC for deterioration in Voice was comparatively precise, the MIC estimates for improvement in Tingling or Numbness and, most markedly, Temperature Tolerance were associated with wide confidence intervals, underlining that these specific estimates are less robust. We had already expected this and we had made a similar observation in our previous study with head and neck cancer patients [7, 8]. We had however not extended the SSQ with more domains because we had started this study before we could determine SIT for the EORTC QLQ-HN43 and we were wary of burdening the patients excessively if the questionnaire was too lengthy. Based on our experiences in both studies, we suggest that future studies take this risk of potentially missing some patients or data due to a longer questionnaire for a chance to obtain better MIC estimates.

Another result is that MIC values were generally lower than MDC values (e.g. for *fatigue*: 8 versus 12 for deterioration, 2 versus 11 for improvement), which again supports the assumption that these two approaches capture different aspects of clinical changes and cannot replace one another [16, 40].

Consistent with previous studies [6], the MIC estimates for improvements over time were generally lower than the corresponding MIC estimates for deteriorations. This suggests that patients are often able to accept certain declines in their QoL and are sensitive to or value even small improvements.

For many of the EORTC scales we examined, the commonly used 10-point rule of thumb estimate would be higher than our MIC values. This would have substantial consequences if the MIC is used to calculate the sample size for a clinical trial. We therefore support the claims of Clarke et al. that trialists should consider adopting contemporary thresholds, rather than legacy thresholds, when using the EORTC QLQ scales as outcomes [30]. We also agree that thresholds derived for a similar patient population should be applied whenever available because SIT are primarily a characteristic of the patient population, not of the instrument.

Of note, the SIT estimates reported here apply to a mixed thyroid cancer population, spanning differentiated, medullary, and anaplastic disease, stages I–IV, and a range of different treatments. We did not derive histology-, stage-, or treatment-specific thresholds, since several subgroups (e.g. anaplastic, poorly differentiated) were too small for stable estimation. Users should therefore treat these values as thresholds for a broad thyroid cancer population rather than as necessarily generalisable to a more homogeneous clinical group. The calculation of subgroup-specific thresholds would be a valuable next step, requiring sufficiently large cohorts. Of note, deriving subgroup-specific thresholds for every clinically, demographically, and culturally meaningful combination would quickly produce tables too unwieldy for practical use. We see this as a direction for future work, ideally supported by an interactive, database-driven approach once a consensus method for deriving and aggregating such granular thresholds is established.

When using the MIC for determining the sample size in a trial, it is important to keep in mind that MIC does not simply equal the difference between arms (which is usually smaller than the MIC). Instead, the (assumed) proportion of improved / deteriorated patients in each arm by at least the MIC can be used to define responders.

Probably the most relevant limitation of our study is that we did not have specific anchors for all EORTC scales, as already outlined above. This made the MIC estimates for the remaining scales less convincing. Further, the average changes in QoL were only moderate and heterogeneous. We had expected a clear deterioration between the start of treatment and a few weeks later, with a clear improvement by half a year later. However, there were also a number of patients who reported improvement after the start of treatment in some domains and also some who experienced deterioration later on. The lack of consistent deterioration after treatment initiation may reflect psychological relief, variability in treatment type and/or intensity, and baseline symptom differences, highlighting the complexity of clinical and QoL trajectories in thyroid cancer. As a result, the MIC analyses suffered from the fact that the proportion of deteriorated patients was below 40% in most domains (the proportions were better regarding improvement). Though we could adjust for this using a formula developed by Terluin and colleagues [39], there is some “noise” in the data that makes the results more difficult to interpret (e.g., MIC of 0).

Moreover, our observation window may not have been long enough to capture the full recovery trajectory for some outcomes, such as parathyroid function, voice, or shoulder function after neck dissection. The MIC values reported here should therefore be understood as specific to this time frame; thresholds at later time points may differ.

Another important limitation is the inability to identify sufficiently strong external anchors for MID estimation using our predefined threshold. While this conservative approach ensured high validity, it may have excluded potentially informative moderate associations. As a consequence, trialists can only rely on the distribution-based estimates for this type of comparison. Moreover, the cohort included heterogeneous thyroid cancer types, treatments, and disease trajectories [55–57]; MIC thresholds may vary across clinical contexts, but this was not explored in the present analysis.

Lastly, although baseline QoL was similar between participation groups, some characteristics (e.g., UICC stage, planned treatment) differed between them; therefore, some degree of attrition bias related to these characteristics cannot be completely excluded.

Our study is the first to have determined SIT for the EORTC QLQ-THY34. It is therefore not possible to compare our SIT values with those of other studies. However, it should be noted that there were considerable differences in the estimates across the various scales. Therefore, researchers must carefully decide which domains are of most interest to them and use the SIT relevant to that endpoint. As other authors have already noted [6, 15, 58], it is unlikely that a single number can serve as a universally applicable threshold for determining SIT across various scales.

Both clinicians and researchers can use the SIT we have derived. They should bear in mind that the estimates serve different purposes: MIC and MDC are benchmarks for interpreting changes over time; MID and MDD are benchmarks for interpreting differences between groups at a given time-point. The MIC can be used for various purposes: (1) as a threshold for determining the number of responders in clinical trials which can add the patient’s perspective to other endpoints; (2) as a threshold for determining the number of responders in groups of patients receiving a specific treatment; (3) to inform patients about the expected effect of the treatment, which can facilitate shared decision-making. Of note, the MIC alone does not indicate how likely a specific treatment is to produce a given change; this would additionally require treatment-specific estimates of the probability and magnitude of QoL change, which are beyond the scope of the present study. The MIC can thus serve not only as a (deterministic) threshold for response but also as a probabilistic estimate for interpreting change scores in terms of the probability that an individual patient has experienced a significant change. Clinicians wishing to use the MIC for individual patients must bear in mind that the estimates were derived from patient groups but are applied to individual patients. This has a consequence: While the thresholds remain the same, the interpretation shifts from proportions to probabilities [59]. The MDC is also a relevant measure of change, but it lacks the patient perspective. It tells us when the observed changes are greater than measurement error. It is derived from reliability estimates and reflects statistical confidence that a change is “real” for a single patient rather than noise. In clinical trials, which primarily test group-level differences, applying the MDC can be misleading because a statistically significant group difference can exist even if most individuals do not exceed MDC, because (a) small consistent changes across many participants can shift the group mean and (b) between-subject variability can mask individual-level “true change”. However, the MDC can act as a lower bound for interpreting meaningful change in clinical trials.

MID and MDD can be used to interpret score differences between patient groups at a given time-point in clinical trials. It should be noted that the estimates are based on a patient average. While the percentage of responders at the group level is likely accurate, each patient may have their own MIC, and therefore some individuals may be misclassified. Additionally, measurement errors contribute to the misclassification of individual patients.

To aid clinical interpretation, we suggest more clearly distinguishing MIC from MDC: while the MIC reflects the smallest change perceived as important by patients, the MDC represents detectable change beyond measurement error. Thus, changes exceeding the MIC may be clinically meaningful even if they do not exceed the MDC, whereas changes exceeding the MDC but not the MIC may be statistically detectable without being relevant from the patient perspective. Conversely, when a patient-reported change exceeds the MIC but remains below the MDC, this cannot be interpreted as certain evidence of a true individual-level change: the magnitude reached is one that patients consider important, but it remains statistically indistinguishable from measurement error for that specific patient. In such cases, clinicians should treat the change as clinically suggestive rather than confirmed, and rely on the probabilistic, group-derived interpretation of the MIC discussed above rather than treating the threshold as a certain individual responder cut-off.

This distinction has a further practical implication for domains scored from few items. Given the EORTC tool’s response format (four ordered categories per item, linearly transformed to a 0–100 scale), the smallest score change an individual patient can register is 100/(3×k) points, where k is the number of items contributing to a domain: 33 points for the eight single-item domains of the EORTC QLQ-THY34, 17 points for the three two-item domains, 11 points for the four three-item domains, and 8 points for the two four-item domains. For several domains, this minimum achievable step exceeds the corresponding MIC estimate — for example, Voice has three items (minimum step 11) but an estimated MIC of 7. In such cases, no single patient can move by an increment as small as the MIC itself; the threshold is therefore best understood as a group-level benchmark — meaningful when averaged or applied probabilistically across many patients — rather than as an individually achievable responder cut-off. As practical guidance: There is no universal MIC applicable across all domains, so researchers should use domain-specific MIC values whenever possible (these values can be found in Table 6). MIC values are generally more informative when the aim is to interpret the clinical importance of change, whereas MDC values remain relevant when assessing whether an individual change exceeds measurement error.

**Table 6:** All SIT estimates combined

		Change				Difference
		Deterioration		Improvement		
		**MIC**	**MDC**	**MIC**	**MDC**	**MDD**
**Specific Anchor**	Exhaustion	**5**		**1**		
	Voice	**13**		**7**		
	Tingling or Numbness	**11**		**2**		
	Temperature Tolerance	**12**		11		
	Global Quality of Life	**2**		**2**		
**Global Anchor**	**EORTC QLQ-THY34**					
	Exhaustion	**3**	12	**1**	13	13
	Discomfort in the Head and Neck	**5**	11	6	9	11
	Voice	**8**	13	**6**	11	12
	Hair Problems	5	10	1	10	10
	Swallowing	**4**	9	4	8	9
	Treatment- and Disease-related Worry	1	8	**0**	8	9
	Tingling or Numbness	5	9	6	9	8
	Worry about Important Others	6	10	**2**	9	11
	Lacking Social Support	5	12	1	13	12
	Dry Mouth	1	12	7	11	12
	Altered Temperature Tolerance	**6**	15	1	15	14
	Body Image	**7**	13	2	14	14
	Shoulder Functioning	4	12	0	10	12
	Joint Pain	5	13	3	14	14
	Cramps	2	15	1	15	14
	Impact on Job or Education	33	14	**2**	14	15
	Rapid Heartbeat	1	11	0	12	11
	**EORTC QLQ-C30**					
	Physical Functioning	**2**	7	2	7	8
	Role Functioning	**3**	14	6	13	13
	Emotional Functioning	**2**	11	**0**	11	11
	Cognitive Functioning	**1**	8	2	8	9
	Social Functioning	**5**	10	**3**	11	11
	Global Health Status/QoL	**2**	10	**2**	9	9
	Fatigue	**8**	12	**2**	11	12
	Nausea and Vomiting	1	10	1	9	7
	Pain	**4**	14	**1**	12	13
	Dyspnoea	27	25	3	13	17
	Insomnia	2	15	3	14	14
	Appetite Loss	**3**	16	1	15	14
	Constipation	17	12	5	12	13
	Diarrhoea	4	11	1	12	9
	Financial Difficulties	6	8	2	9	11

*Note: SIT = Score interpretation thresholds*,* MIC = Minimal important change*,* MDC = Minimal detectable change*,* MDD = Minimal detectable difference. This table combines the estimates from* Tables 3 and 4, and 5. MIC with robust correlation with the anchor (*r* > 0.15) are marked with bold printing

From a clinical and research perspective, our findings provide practical thresholds for interpreting score differences when using the EORTC core questionnaire and its thyroid cancer module in thyroid cancer patients. The availability of domain-specific MIC values facilitates more precise identification of responders in clinical trials and supports patient-centred decision-making in routine care. These thresholds should be applied in the context of the specific patient group and clinical setting, while also taking statistical uncertainty into account [59].

## Conclusions

Our results underscore that no single MIC or MDC can be applied to all scales of the EORTC QLQ-THY34. Moreover, the MICs for deterioration appear to be larger than those for improvement. It should be noted that the precision of these estimates varies considerably across domains; some MIC estimates are based on weaker anchor correlations and wide confidence intervals and should therefore be interpreted with caution.

The results may help researchers and clinicians interpret the scores of the EORTC QLQ-C30 and the EORTC QLQ-THY34 in patients with thyroid cancer and plan clinical trials. We were able to use domain-specific anchor values for five domains and found that domain-specific anchor values were indeed superior to a global anchor value when calculating MICs. Therefore, future research should use domain-specific anchor values whenever possible.

## Data Availability

The data of this study are stored in the EORTC data repository and can be accessed by other researchers.
